# Temporal trends in the systemic inflammatory response syndrome, sepsis, and medical coding of sepsis

**DOI:** 10.1186/s12871-015-0148-z

**Published:** 2015-11-24

**Authors:** Benjamin S. Thomas, S. Reza Jafarzadeh, David K. Warren, Sandra McCormick, Victoria J. Fraser, Jonas Marschall

**Affiliations:** 1 Division of Infectious Diseases, Department of Medicine, Washington University School of Medicine, 660 South Euclid Avenue, Campus Box 8051, St. Louis, MO 63110 USA; 2 Department of Medicine, John A. Burns School of Medicine, 651 Ilalo Street, Honolulu, 96813 HI USA; 3 Center for Clinical Excellence, BJC HealthCare, 4901 Forest Park Avenue, St. Louis, 63108 MO USA; 4 Department of Infectious Diseases, Bern University Hospital and University of Bern, Friedbühlstrasse 51, CH-3010 Bern, Switzerland

## Abstract

**Background:**

Recent reports using administrative claims data suggest the incidence of community- and hospital-onset sepsis is increasing. Whether this reflects changing epidemiology, more effective diagnostic methods, or changes in physician documentation and medical coding practices is unclear.

**Methods:**

We performed a temporal-trend study from 2008 to 2012 using administrative claims data and patient-level clinical data of adult patients admitted to Barnes-Jewish Hospital in St. Louis, Missouri. Temporal-trend and annual percent change were estimated using regression models with autoregressive integrated moving average errors.

**Results:**

We analyzed 62,261 inpatient admissions during the 5-year study period. ‘Any SIRS’ (i.e., SIRS on a single calendar day during the hospitalization) and ‘multi-day SIRS’ (i.e., SIRS on 3 or more calendar days), which both use patient-level data, and medical coding for sepsis (i.e., ICD-9-CM discharge diagnosis codes 995.91, 995.92, or 785.52) were present in 35.3 %, 17.3 %, and 3.3 % of admissions, respectively. The incidence of admissions coded for sepsis increased 9.7 % (95 % CI: 6.1, 13.4) per year, while the patient data-defined events of ‘any SIRS’ decreased by 1.8 % (95 % CI: −3.2, −0.5) and ‘multi-day SIRS’ did not change significantly over the study period. Clinically-defined sepsis (defined as SIRS plus bacteremia) and severe sepsis (defined as SIRS plus hypotension and bacteremia) decreased at statistically significant rates of 5.7 % (95 % CI: −9.0, −2.4) and 8.6 % (95 % CI: −4.4, −12.6) annually. All-cause mortality, SIRS mortality, and SIRS and clinically-defined sepsis case fatality did not change significantly during the study period. Sepsis mortality, based on ICD-9-CM codes, however, increased by 8.8 % (95 % CI: 1.9, 16.2) annually.

**Conclusions:**

The incidence of sepsis, defined by ICD-9-CM codes, and sepsis mortality increased steadily without a concomitant increase in SIRS or clinically-defined sepsis. Our results highlight the need to develop strategies to integrate clinical patient-level data with administrative data to draw more accurate conclusions about the epidemiology of sepsis.

## Background

Sepsis is a clinical syndrome characterized by a dysregulated systemic inflammatory response to an infection that may be complicated by one or more organ dysfunctions, which is termed severe sepsis. Sepsis is a significant cause of morbidity and mortality among hospitalized patients. It is estimated that upwards of one million persons in the United States develop sepsis each year, with an associated mortality of approximately 30 % [1, 2]. The financial costs associated with sepsis care have been estimated to be between $20,000 and $30,000 per case [1]. Estimates of the frequency of sepsis are important for public health strategic planning and prioritization of resources to reduce the burden of disease; however, most sepsis surveillance reports have relied on inpatient administrative claims data. National efforts are focused on improving outcomes in patients with sepsis and hospital associated sepsis rates have been proposed as a quality of care indicator [3, 4]. Modest success reducing sepsis mortality was achieved in some studies with the implementation of the Surviving Sepsis Campaign bundle; however, mortality due to sepsis is still high [5, 6].

Definitions for the systemic inflammatory response syndrome (SIRS) and sepsis were originally agreed upon at a consensus conference in 1991 and have been revisited several times to refine how the continuum of sepsis is described [7]. Over the last 20 years, several studies reported national trends in the incidence of sepsis, using *International Classification of Diseases*, Ninth Revision, Clinical Modification (ICD-9-CM) discharge diagnosis codes using large inpatient hospital administrative claims datasets [2, 8, 9]. Based on this type of data, estimates of the incidence of severe sepsis, clinically defined as syndrome of infection plus end-organ dysfunction, have ranged from 303 to 1,074 cases per 100,000 population [1]. The incidence of severe sepsis in these studies increased between 8 · 2 to 13 · 3 % annually in the last 15 to 20 years [2, 8, 10]. A major limitation of the underlying data in these studies is that diagnosis codes have not been correlated with clinical measurements, such as vital sign measurements, laboratory tests, and microbiological data. The few epidemiological studies of sepsis that included clinical information did not examine trends over time and were limited by factors such as short study durations, restriction to intensive care unit (ICU) patients, and single center design [11–13].

Given the ongoing debate surrounding the incidence of sepsis in the United States and potential changes in coding practices over time, the purpose of this study was to characterize temporal trends in sepsis incidence, based on administrative claims data at a large, Midwestern, tertiary care hospital, and compare these administrative claims data with clinical data in order to better understand the trends in SIRS, sepsis and sepsis mortality over time.

## Methods

### Study setting and patient population

Barnes-Jewish Hospital (BJH) is a 1250-bed, academic, tertiary-care center located in St. Louis, Missouri, USA, which is affiliated with Washington University School of Medicine. Approximately 50,000 patients are admitted to BJH each year. BJH has six adult intensive care units, plus newborn and special care nurseries. We included all adults (≥18 years old) admitted to BJH within the first week of each month between January 1, 2008 and December 31, 2012. Data on individual patients admitted to the hospital during this time frame were obtained from the medical informatics system, a relational database which includes all vital sign measurements, laboratory tests, blood culture results, admission and discharge dates, discharge ICD-9-CM diagnoses codes, and discharge status (e.g., death, discharge to home).

### Definitions

#### Clinical parameters

The systemic inflammatory response syndrome (SIRS) was based on the criteria by Bone et al. and was defined as the presence of at least two of four criteria occurring on a given calendar day - heart rate (>90 beats/min); respiratory rate (>20 breaths/min); temperature (>38 · 3 or <36 °C); and white blood cell count (>12,000 or <4,000 microL^−1^) [3, 7]. In order to minimize the proportion of patients that transiently met SIRS criteria (e.g., a post-operative patient in pain with a single measurement of tachypnea and tachycardia), at least two subsequent measurements for any of the three vital sign criteria had to be outside the normal range on a given calendar day in order to be counted towards SIRS (i.e., patients who had only a single out-of-range measurement would therefore not be counted as SIRS). Although blood samples for laboratory testing may drawn once or many times daily, we accepted a single abnormal white blood cell count measurement in a calendar day to define SIRS. We defined patients with “any SIRS” as meeting the above definition of SIRS on at least a single calendar day during the hospitalization and patients with “multi-day SIRS” as meeting SIRS criteria on three or more (consecutive or non-consecutive) calendar days. “Any SIRS” and “multi-day SIRS” are not mutually exclusive.

We determined if patients had “SIRS plus hypotension”, if they had a systolic blood pressure <90 mmHg within a calendar day of the SIRS event. SIRS plus bacteremia (within a calendar day of the SIRS event) was defined as “sepsis” by clinical parameters. SIRS plus hypotension combined with bacteremia (within a calendar day of the SIRS event) was classified as “severe sepsis” by clinical parameters (Table 1). It should be noted that these definitions are reflecting sepsis or severe sepsis with confirmed bacteremia, which represents a fraction of all sepsis cases.

**Table 1:** Variable explanations

Variable	Comment	Type
SIRS	Uses the Surviving Sepsis Campaign [3] definition of the systemic inflammatory response syndrome. At least two vital sign measurements were required outside the range on a given calendar day (for each of the three vital signs: temperature, heart rate, and respiratory rate).	Patient-level
Sepsis	Defined by one of the following ICD-9 codes: 995 · 91, 995 · 92, 785 · 52	Administrative
Sepsis (Angus)	Defined using the ICD-9 codes outlined in Angus et al., *Crit Care Med*. 2001;29 (7):1303–1310	Administrative
Sepsis (Dombrovskiy)	Defined using the ICD-9 codes outlined in Dombrovskiy et al., *Crit Care Med*. 2007;35 (5):1244–1250	Administrative
Sepsis	SIRS plus bacteremia (within a calendar day of SIRS event)	Patient-level
Severe sepsis	SIRS plus Hypotension (Systolic blood pressure <90 mmHg, occurring within a calendar day of the SIRS event) + Bacteremia	Patient-level
SIRS plus hypotension	SIRS plus Hypotension	Patient-level
Any SIRS	SIRS on a single calendar day during the hospitalization	Patient-level
Multi-day SIRS	SIRS on 3 or more calendar days	Patient-level
Bacteremia	First positive blood culture within a calendar day of a SIRS event. Common skin contaminants were excluded (Coagulase-negative staphylococci, *Micrococcus* spp, *Propionibacterium acnes*, *Bacillus* spp, *Corynebacterium* spp.).	Patient-level

Bacteremia was defined as the first positive blood culture within a calendar day of a SIRS event. Common skin contaminants were excluded (i.e., Coagulase-negative staphylococci, *Micrococcus spp*., *Propionibacterium spp.*, *Bacillus spp*., *Corynebacterium spp*.).

### Medical coding

We defined sepsis as occurring during a patient’s hospitalization if the ICD-9-CM discharge diagnosis codes included sepsis (995 · 91), severe sepsis (995 · 92), or septic shock (785 · 52). We also compared this coding algorithm for sepsis to two previously published sepsis coding algorithms by Angus et al. and Dombrovskiy et al., which both used administrative claims data [2, 8]. Finally, the Charlson comorbidity index, a weighted sum of 19 comorbid conditions based on ICD-9-CM codes, was calculated for each patient [14].

### Statistical analyses

We calculated monthly incidence rates for sepsis (by ICD-9-CM codes), sepsis (SIRS plus bacteremia), severe sepsis (SIRS plus hypotension plus bacteremia), SIRS plus hypotension, any SIRS, multi-day SIRS, and bacteremia per 1,000 patient-days (Table 3). Mortality rates associated with any SIRS, multi-day SIRS, sepsis, and all-cause mortality as well as any SIRS, multi-day SIRS, and sepsis fatality rates were also determined. The proportion of patients with a Charlson comorbidity score ≥5 in each month (to understand if the inpatient population’s level of medical complexity changed) and monthly average number of discharge diagnosis codes were also calculated. Linear temporal-trend models were fitted to the natural logarithm of each rate through dynamic regression modeling. This was done to account for substantial correlation in the corresponding residuals over time. We accounted for the autocorrelation of residuals through autoregressive integrated moving average (ARIMA) modeling, using regression models with ARIMA errors.

Annual percentage change (APC) in rates and proportions was calculated from the estimated trends as: APC = [e^intercept + coefficient (month + 12)^ - e^intercept + coefficient (month)^] / e^intercept + coefficient (month)^ * 100, where intercept and coefficient are estimated from the corresponding models for each series [15]. All analyses were performed in R software (R Foundation for Statistical Computing, Vienna, Austria) [16].

The institutional review board of the Washington University Human Research Protection Office (IRB# 201304071) approved the research protocol with a waiver of written informed consent.

## Results

### Patient characteristics/demographics

The total number of patients who were admitted during the first week of each month from 2008 to 2012 was 62,261 and represented 327,205 patient-days. The median age (years, interquartile range) for all study patients, patients with any SIRS, multi-day SIRS, and for those patients coded for sepsis were 55 · 0 (41, 67), 56 · 0 (42, 68), 59 · 0 (46, 69), and 60 · 0 (50, 71), respectively. The racial composition was predominantly white (62 · 6 %) and African American (34 · 4 %) with a minority being Asian/Pacific Islander or Native American (0 · 9 %). The median length of hospital stay for all the study patients was 3 · 0 days (range, 2 · 0, 6 · 0), and was progressively longer for patients with any SIRS, multi-day SIRS, and for patients coded for sepsis (Table 2). Admission to an ICU during the hospital course (at any time) occurred in 16 · 7 % of all patients admitted to the hospital. A majority (74 %) of patients who were subsequently coded for sepsis were admitted to an ICU (at any time) during the hospital course.

**Table 2:** Characteristics of patients in the study population

Characteristic	All *N* = 62,261	Any SIRS *N* = 21,962	Multi-day SIRS *N* = 10,759	SIRS plus bacteremia *N* = 1,412	SIRS plus hypotension *N* = 2,026	SIRS plus hypotension and bacteremia *N* = 193	Coded for sepsis *N* = 2,062
Age [IQR]	55.0 [41.0, 67.0]	56.0 [42.0, 68.0]	59.0 [46.0, 69.0]	58.5 [48.0, 68.0]	59.0 [48.0, 70.0]	59.0 [49.5, 68.0]	60.0 [49.75, 71.0]
Male sex (%)	28,506 (45.8)	10,526 (47.9)	5,515 (51.3)	766 (54.2)	1,006 (49.7)	100 (51.8)	1,107 (53.7)
Race							
- White (%)	39,004 (34.4)	14,382 (65.5)	7,453 (69.3)	910 (64.4)	1,467 (72.4)	123 (63.7)	1,370 (66.4)
- African American, Black (%)	21,420 (34.4)	6,943 (31.6)	3,030 (28.2)	459 (32.5)	493 (24.3)	59 (30.6)	626 (30.4)
- Asian, PI/NA (%)	580 (0.9)	211 (1.0)	94 (0.9)	19 (1.4)	25 (1.2)	4 (2.0)	27 (1.3)
- Other, unknown (%)	1,257 (2.0)	426 (1.9)	182 (1.7)	24 (1.7)	41 (2.0)	9 (4.6)	39 (1.9)
Length of stay [IQR]	3.0 [2.0, 6.0]	6.0 [3.0, 11.0]	10.0 [6.0, 17.0]	13.0 [6.0, 25.0]	7.0 [4.0, 13.0]	13.0 [5.0, 24.5]	11.0 [5.0, 21.25]
ICU admission (%)	10,377 (16.7)	8,193 (37.3)	5,634 (52.4)	815 (57.7)	1,267 (62.5)	151 (78.2)	1,521 (73.8)
In-hospital all-cause mortality (%)	1,715 (2.8)	1,535 (7.0)	1,023 (9.5)	292 (20.7)	328 (16.2)	58 (30.1)	654 (31.7)

SIRS: Systemic Inflammatory Response Syndrome; sepsis ICD-9-CM codes: 995 · 91, 995 · 92 and 785 · 52; IQR: Inter-quartile range for the corresponding median; PI/NA: Pacific Islander, Native American; ICU: intensive care unit

### Temporal trends for SIRS and sepsis

The incidence of sepsis based on administrative coding increased at a statistically significant annual rate of 9 · 7 % (95 % Confidence Interval (CI): 6 · 1, 13 · 4) during the study period, similar to the coding algorithms established by Angus and Dombrovskiy, which showed significant upward trends with an annual percent change of 5 · 8 % (95 % CI: 4 · 0, 7 · 5) and 5 · 5 % (95 % CI: 3 · 4, 7 · 6), respectively. In contrast, the annual percent change for any SIRS was −1 · 8 % (95 % CI: −3 · 2, −0 · 5) and the rate of multi-day SIRS did not significantly change at all (−0 · 1 %; 95 % CI: −1 · 7, 1 · 5) (Table 3).

**Table 3:** Annual percent change and estimates of linear temporal trends for (the natural logarithm of) rates of sepsis, SIRS, bacteremia, sepsis-related mortality, SIRS-related mortality, overall hospital mortality and average coding

	Trend coefficient (95 % CI)	Annual percent change (95 % CI)
Coding variables		
Sepsis	0 · 0077 (0 · 0049, 0 · 0105)^a^	9 · 7 (6 · 1, 13 · 4)
Sepsis (Angus)	0 · 0047 (0 · 0033, 0 · 0060)^a^	5 · 8 (4 · 0, 7 · 5)
Sepsis (Dombrovskiy)	0 · 0044 (0 · 0028, 0 · 0061)^a^	5 · 5 (3 · 4, 7 · 6)
Sepsis mortality	0 · 0070 (0 · 0015, 0 · 0125)^a^	8 · 8 (1 · 9, 16 · 2)
Sepsis mortality (Angus)	0 · 0080 (0 · 0015, 0 · 0145)^a^	10 · 0 (1 · 8, 18 · 9)
Sepsis mortality (Dombrovskiy)	0 · 0051 (−0 · 0007, 0 · 0110)	6 · 3 (−0 · 8, 14 · 1)
Sepsis fatality	−0 · 0004 (−0 · 0065, 0 · 0057)	−0 · 5 (−7 · 5, 7 · 0)
Sepsis fatality (Angus)	0 · 0026 (−0 · 0019, 0 · 0072)	3 · 2 (−2 · 2, 9 · 0)
Sepsis fatality (Dombrovskiy)	0 · 0010 (−0 · 0046, 0 · 0066)	1 · 2 (−5 · 4, 8 · 2)
Sepsis & Charlson ≥5	0 · 0073 (0 · 0035, 0 · 0111)^a^	9 · 1 (4 · 3, 14 · 2)
Sepsis & ICU	0 · 0075 (0 · 0046, 0 · 0104)^a^	9 · 4 (5 · 7, 13 · 3)
ICU & Charlson ≥5	−0 · 0011 (−0 · 0051, 0 · 0029)	−1 · 3 (−5 · 9, 3 · 6)
Average coding	0 · 0128 (0 · 0110, 0 · 0147)^a^	16 · 6 (14 · 1, 19 · 3)
Clinical variables		
Sepsis	−0 · 0049 (−0 · 0079, −0 · 0020)^a^	−5 · 7 (−9 · 0, −2 · 4)
Severe sepsis	−0 · 0075 (−0 · 0112, −0 · 0038)^a^	−8 · 6 (−4 · 4, −12 · 6)
SIRS plus hypotension	−0 · 0085 (−0 · 0146, −0 · 0025)^a^	−9 · 7 (−16 · 1, −2 · 9)
Any SIRS	−0 · 0015 (−0 · 0027, −0 · 0004)^a^	−1 · 8 (−3 · 2, −0 · 5)
Multi-day SIRS	−0 · 0001 (−0 · 0014, 0 · 0013)	−0 · 1 (−1 · 7, 1 · 5)
Bacteremia	−0 · 0055 (−0 · 0083, −0 · 0026)^a^	−6 · 3 (−9 · 5, −3 · 1)
All-cause mortality	−0 · 0005 (−0 · 0035, 0 · 0025)	−0 · 6 (−4 · 1, 3 · 0)
Sepsis mortality	−0 · 0063 (−0 · 0098, −0 · 0027)^a^	−7 · 3 (−11 · 1, −3 · 2)
Severe sepsis mortality	−0 · 0019 (−0 · 0055, 0 · 0018)	−2 · 2 (−6 · 4, 2 · 1)
SIRS plus hypotension mortality	−0 · 0111 (−0 · 0260, 0 · 0037)	−12 · 5 (−26 · 8, 4 · 6)
Any SIRS mortality	0 · 0005 (−0 · 0027, 0 · 0037)	0 · 6 (−3 · 2, 4 · 5)
Multi-day SIRS mortality	0 · 0013 (−0 · 0035, 0 · 0061)	1 · 5 (−4 · 2, 7 · 5)
Sepsis fatality	0 · 0016 (−0 · 0047, 0 · 0079)	1 · 9 (−5 · 5, 10 · 0)
Severe sepsis fatality	−0 · 0048 (−0 · 0377, 0 · 0280)	−5 · 6 (−36 · 4, 40 · 0)
SIRS plus hypotension fatality	−0 · 0051 (−0 · 0104, 0 · 0003)	−5 · 9 (−11 · 8, 0 · 4)
Any SIRS fatality	0 · 0021 (−0 · 0011, 0 · 0052)	2 · 5 (−1 · 3, 6 · 4)
Multi-day SIRS fatality	0 · 0007 (−0 · 0027, 0 · 0041)	0 · 8 (−3 · 2, 5 · 0)

^a^The confidence interval does not include 0

Note: ICU – Intensive Care Unit; SIRS – Systemic Inflammatory Response Syndrome; Sepsis (coding variable) = 995 · 91, 995 · 92, 785 · 52; Sepsis (clinical variable) = SIRS plus bacteremia; Severe sepsis (clinical variable) = SIRS plus hypotension plus bacteremia

Sepsis (SIRS plus bacteremia) decreased at a statistically significant rate of 5 · 7 % (−5 · 7; 95 % CI: −9 · 0, −2 · 4) over the entire study period. SIRS plus hypotension and severe sepsis, which were both also calculated using patient-level data, decreased at statistically significant rates of 9 · 7 % (−9 · 7; 95 % CI: −16 · 1, −2 · 9) and 8 · 6 % (−8 · 6; 95 % CI: −4 · 4, −12 · 6), respectively, from August 2009 to December 2012 (Note: blood pressure readings were not available in the informatics database between January 2008 and July 2009).

### Temporal trends in mortality

**Fig. 1:**
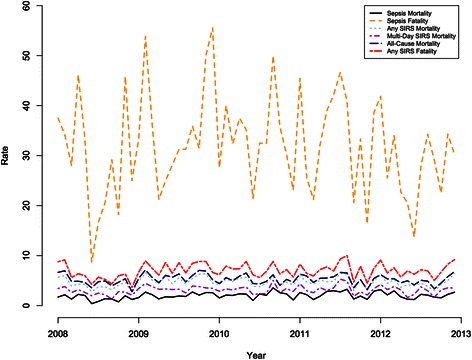
Rates (per 1,000 patient-days) for Sepsis, Any SIRS, Multi-day SIRS, and All-Cause Mortality and Ratios (%) for Sepsis and SIRS Fatality

All-cause hospital mortality among the entire patient sample was 2 · 8 %. The mortality associated with sepsis (by ICD-9 coding) showed an annual increase of 8 · 8 % (95 % CI: 1 · 9, 16 · 2) during the study period. The same was found for sepsis mortality as defined by the Angus method, which showed an increase of 10 · 0 % (95 % CI: 1 · 8, 18 · 9) per year. Sepsis mortality (defined using clinical criteria) showed a statistically significant decrease at 7 · 3 % per year (95 % CI: (−11 · 1, −3 · 2). All-cause hospital mortality was highest for those coded for sepsis (31 · 7 %), followed by those with multi-day SIRS (9 · 5 %) and any SIRS (7 · 0 %). The sepsis case fatality rate did not show a statistically significant change over time using all three administrative data based algorithms of sepsis (ICD-9, Angus, and Dombrovskiy) or by clinical criteria (Fig. 1). SIRS plus hypotension mortality, severe sepsis mortality, and the corresponding case fatality rates did not change significantly over the study period. Additionally, the SIRS mortality rates and SIRS case fatality rates did not show a statistically significant change over time (Table 3).

### Temporal trends in coding, bacteremia, and severity of illness

**Fig. 2:**
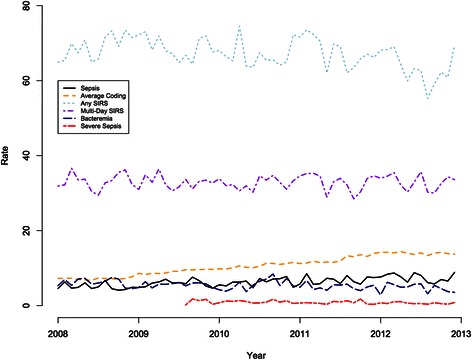
Rates (per 1,000 patient-days) for Sepsis, Severe Sepsis, Any SIRS, Multi-day SIRS, Bacteremia, and Average Coding

The average number of discharge diagnosis codes per admission increased by 16 · 6 % (95 % CI: 14 · 1, 19 · 3) during each year of the study period (Fig. 2). The subset of patients with a Charlson comorbidity score ≥5 did not change significantly over the study period while the proportion of patients with a Charlson score ≥5 and who were coded for sepsis increased by 9 · 1 % per year (95 % CI: 4 · 3, 14 · 2). Additionally, sepsis coding among ICU patients increased at 9 · 4 % per year (95 % CI: 5 · 7, 13 · 3). The rate of bacteremia, among all patients, decreased steadily by 6 · 3 % (−6 · 3; 95 % CI: −9 · 5, −3 · 1) annually.

## Discussion

At our institution, the incidence of sepsis, based on administrative claims data, increased by 9 · 7 % annually during the study period, which is consistent with other studies that have used administrative claims data to report sepsis incidence [2, 8, 9]. However, we did not see a concomitant increase in patient-level variables associated with sepsis. For example, there was no significant increase in the systemic inflammatory response syndrome (SIRS); SIRS events even decreased slightly over the study period. The rate of positive blood cultures (bacteremia being one of the predominant causes of sepsis) also trended downwards over time. Moreover, the patient-level information used as a *proxy marker* for severe sepsis (i.e., SIRS plus hypotension) and employed in our definition of *sepsis* (SIRS plus bacteremia) and *severe sepsis* (SIRS plus bacteremia and hypotension) yielded decreasing rates over time. We hypothesize that increasingly accurate documentation of sepsis in the medical record has resulted in improved administrative coding for sepsis and other medical conditions. There has also been an overall increase in the number of discharge diagnosis codes among hospitalized patients, which may reflect better documentation and coding and/or increasing prevalence of multiple comorbidities among hospitalized patients. In addition, we suspect that the increase in sepsis mortality among coded patients reflects more accurate documentation of severity of illness and sepsis coding practices than in the past.

Administrative claims data are a relatively cost effective and readily available means of determining population incidence and performing long-term surveillance for many diseases. However, because administrative claims data are primarily used for billing and not for surveillance or research, these data are susceptible to variation in physician documentation and changes in coding practices. The quality and accuracy of physician documentation may vary considerably and has therefore become the subject of quality improvement initiatives, known as *clinical documentation improvement* (CDI), a [17]. It has been demonstrated that patient admissions due to severe sepsis and septic shock are often under-coded because of inadequate documentation [18]. Also, several studies have reported that the sensitivity and specificity of various coding abstraction methods perform poorly compared to structured chart review [19–21].

While not focusing on sepsis specifically, studies of other medical conditions have found that administrative data coding practices change over time. A recent study on hospitalizations and mortality in patients with pneumonia highlights how administrative claims coding is susceptible to shifts in coding practices. Using the National Inpatient Sample to examine temporal trends in diagnostic coding for a principal diagnosis of pneumonia, sepsis with a secondary diagnosis of pneumonia, or respiratory failure with a secondary diagnosis of pneumonia, Lindenauer et al. found that the supposed improvement in mortality from pneumonia did not reflect improved patient outcomes but rather was a function of variations in coding practices [22]. The authors aptly noted in their discussion that the rapid increase in sepsis and severe sepsis begs the question as to whether the phenomenon of changing trends in coding they saw in pneumonia is also happening more broadly with sepsis. Our findings suggests this is the case, since we saw a steady rate of increase in sepsis coding without a concurrent increase in clinical events such as SIRS and bacteremia that would be expected if there were a true increase in sepsis incidence.

Our study is limited by its single center design, a relatively short study period, and the lack of complete data for blood pressure measurements in one of the study years. Additionally, we cannot determine the reasons behind increased medical coding for sepsis. Coding practices are likely driven by a variety of factors. Administrative claims data, primarily a tool for billing, rely on physician documentation, utilize surrogate markers (i.e., the codes) for diseases and are not directly linked to more comprehensive patient-level clinical data. The increased awareness of sepsis syndromes and international efforts lead by the Surviving Sepsis Campaign to improve sepsis care may have heightened awareness and changed physician documentation practices [3]. One consequence of this is that longitudinal studies conducted in the last decade, which utilized administrative claims data to define sepsis, may not accurately reflect the true epidemiology of community- or healthcare-associated sepsis. Growing pressures on hospital quality measurements, financial incentive programs (pay-for-performance), and the increase in electronic health records may have resulted in better documentation of severity of illness by physicians and hospitals [23, 24].

## Conclusions

In conclusion, administrative claims data coding practices likely shift with changes in healthcare regulations and variation in physician documentation. Coding for sepsis, measured by administrative claims data, has increased over time; however, in our institution the underlying rate of SIRS, SIRS plus hypotension, sepsis, and severe sepsis, as measured using clinical data has remained stable. Administrative data therefore do not reflect clinical trends very well and our results highlight the need to develop strategies to integrate clinical patient-level data with administrative data to draw more accurate conclusions about the epidemiology of sepsis.

## Abbreviations

APC: annual percent change
ARIMA: autoregressive integrated moving average modeling
BJH: Barnes-Jewish Hospital
CDI: clinical documentation improvement
ICD-9-CM: international classification of diseases, ninth revision, clinical modification
ICU: intensive care unit
SIRS: systemic inflammatory response syndrome
